# Characterization of the mitochondrial complete genome of Korean indigenous catfish, *Liobagrus hyeongsanensis* (Siluriformes: Amblycipitidae)

**DOI:** 10.1080/23802359.2021.1947917

**Published:** 2021-07-19

**Authors:** Philjae Kim, Hyeongsu Kim, Suhwan Kim

**Affiliations:** a Division of Ecological Conservation, Bureau of Ecological Research, National Institute of Ecology, Choongnam, Korea; b Advanced aquaculture research center, National Institute of Fisheries Science, Korea

**Keywords:** Korean indigenous catfish, mitochondrial genome, *Liobagrus hyeongsanensis*, molecular phylogenetic analysis

## Abstract

*Liobagrus hyeongsanensis*, Korean indigenous catfish, was reported as a new species in 2015. The complete mitochondrial DNA sequence of *L*. *hyeongsanensis* was sequenced by next-generation sequencing (NGS) analysis. The mitochondrial genome was assembled with 16,529 bp in length and encoded 13 protein-coding genes (PCGs), 22 tRNAs, two rRNAs, and one control region (D-loop). Also, the gene structures such as gene order and content were totally identical with the congeneric species. Molecular phylogenetic analysis determined the taxonomical position of *L*. *hyeongsanensis* in species level among the genus *Liobagrus*.

Genus *Liobagrus* inhabiting in South Korea is composed for five species, *L. andersoni*, *L. obesus*, *L. mediadiposalis*, *L. somjinensis*, and *L. hyeongsanensis* (Park and Kim 2010; Kim et al. 2015). Among these species, the complete mitochondrial genomes were analyzed except *L. hyeongsanensis* Kim et al. 2015 (Kartavtsev et al. 2007; Lee et al. 2016; Park et al. 2017; Kim et al. 2020). We analyzed the complete mitochondrial sequence of *L. hyeongsanensis* in this study and determined the molecular phylogenetic relationship between all of *Liobagrus* species from South Korea. Also, we examined the genome structure and characteristics, gene order and nucleotide base composition, and these results contributed to identify molecular features of the *Liobagrus* species.

*Liobagrus hyeongsanensis* specimen (voucher code: NIE-FI00001) used in mitochondrial genome analysis was collected from Buk River, Hwangnyong-dong, Gyeongju-si (35°51′10.03″N, 129°13′00.00″E) on 10 January 2021. A voucher specimen deposited at National Institute of Ecology (Seocheon, Korea), and mitochondrial DNA (mt-DNA) was stored in freezer (manager: Philjae Kim, swubio@naver.com, Seochen, Korea). Mitochondria was isolated from caudal fin using the Qproteome^®^ Mitochondria Isolation Kit (QIAGEN, Hilden, Germany), and mt-DNA (NIE-DN00001) was extracted from mitochondria sample using DNeasy Blood and Tissue DNA isolation kit (QIAGEN). The extracted mt-DNA was stored in National Institute of Ecology until used. For NGS analysis, we obtained the PCR product from mt-DNA by using REPLI-g Mitochondrial DNA kit (QIAGEN). The library sample was prepared for sequencing analysis using QIAseq FX single cell DNA library kit (QIAGEN). The sequencing analysis was conducted by Illumina Hi-Seq 2500 platform (San Diego, CA, USA) in GnC Bio Co. (Daejeon, South Korea). The treatments of raw data, such as trimming, assembly and gene arrangement, were performed by using Geneious Prime 2021.1 (Biomatters, Auckland, New Zealand). The tRNA genes annotation and secondary structures were examined using tRNAscan-SE 2.0 (Chan and Lowe 2019).

The mitochondrial genome of *L. hyeongsanensis* (MZ066608) was 16,529 bp in length and composed of 13 PCGs, 22 tRNAs, two rRNAs, and one control region (D-loop). The gene order was completely identical as other *Liobagrus* species (Kartavtsev et al. 2007; Lee et al. 2016; Park et al. 2017; Kim et al. 2020). The nucleotide base composition was examined as 30.1% A, 25.3% T, 28.5% C, and 16.0% G. As with congeneric species, COI has initiation codon with ‘GTG’, and 12 of remainder PCGs started with ‘ATG.’ The six PCGs (ND2, COX2, COX3, ND3, ND4 and CytB) were used incomplete terminal codon ‘T––.’ The five PCGs (COX1, ATP8, ATP6, ND4L and ND5) and two PCGs (ND1 and ND6) have ‘TAA’ and ‘TAG’ as terminal codon, respectively.

For determining the molecular phylogenetic relationship among the Korean *Liobagrus* species, we obtained the mitochondrial complete sequences of genus *Liobagrus* deposited in GenBank of NCBI (http://www.ncbi.nlm.nih.gov). All of the sequences were aligned with Geneious Prime 2021.1 (https://www.geneious.com). The dataset for phylogenetic analysis composed of 13 PCGs of a total of 36 Siluriformes mitochondrial genome. The maximum likelihood (ML) analysis with 1000 replicates conducted using PhyML 3.1, and best fit model was estimated by jModel Test with GTR + I + G substitution model (Guindon and Gascuel 2003; Guindon et al. 2010; Darriba et al. 2012). According to ML analysis, *L. hyeongsanensis* was placed on *Liobagrus* clade with congeneric species, and all of the family groups were formed monophyletic clade (Figure 1). These result showed the phylogenetic status based on molecular and morphological evidences is stable in order Siluriformes. Our study would be a contributor for establishing the more precise phylogenetic relationship of Siluriformes.

**Figure 1.**
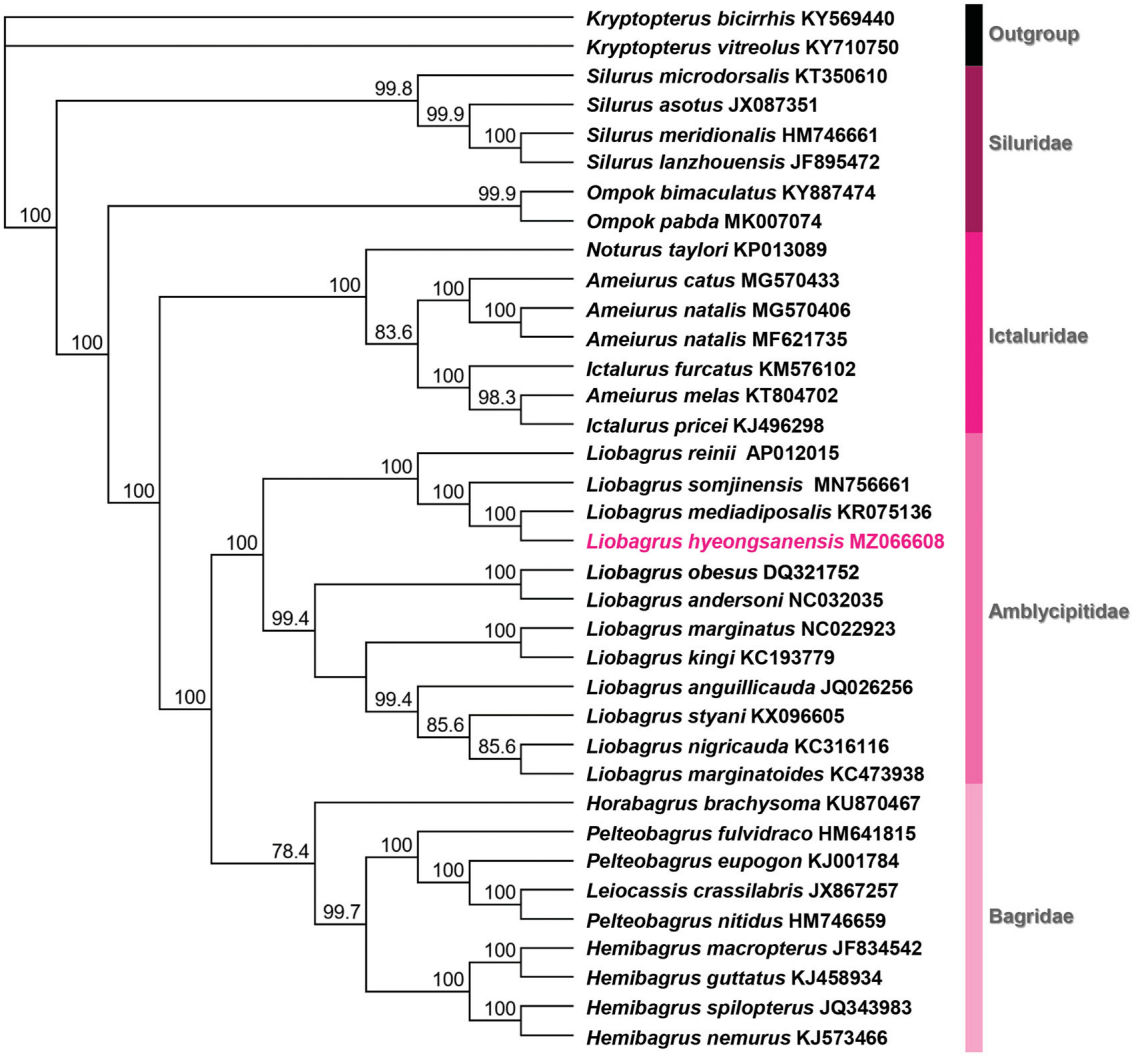
The molecular phylogenetic position of *Liobagrus hyeongsanensis*. The maximum likelihood tree was constructed with GTR + I+G based on 13 PCG sequences 36 Siluriformes species, including *L*. *hyeongsanensis* (MZ066608). Bootstrap support values are indicated on each node as >70.

## Data Availability

The genome sequence data that support the findings of this study are openly available in GenBank of NCBI at (https://www.ncbi.nlm.nih.gov/) under the accession no. MZ066608. The associated BioProject, SRA and Bio-Sample numbers are PRJNA717732, SRR14151707 and SAMN18509289 respectively.
